# Phenylethanol Glycoside from *Cistanche tubulosa* Attenuates BSA-Induced Liver Fibrosis in Rats by Modulating the Gut Microbiota–Liver Axis

**DOI:** 10.3390/ph17091149

**Published:** 2024-08-30

**Authors:** Xinxin Qi, Hongguang Sun, Jincun Liu, Meili Cong, Xinxuan Zhang, Yuxin Yan, Zhaolin Xia, Tao Liu, Jun Zhao

**Affiliations:** 1 School of Public Health, Xinjiang Medical University, Urumqi 830011, China; xjmuqxx@163.com (X.Q.); sunhongguang78@aliyun.com (H.S.); ljc19111@163.com (J.L.); congmeili@126.com (M.C.); 18835454567@163.com (X.Z.); 19025782260@163.com (Y.Y.); zlxia@shmu.edu.cn (Z.X.); 2 Animal Laboratory Center, Xinjiang Medical University, Urumqi 830017, China; 3 Xinjiang Key Laboratory for Uighur Medicine, Institute of Materia Medica of Xinjiang, Urumqi 830004, China

**Keywords:** *Cistanche tubulosa*, phenylethanol glycosides, liver fibrosis, gut microbiota, fecal microbiota transplantation

## Abstract

This study aimed to investigate the effect of phenylethanol glycoside from *Cistanche tubulosa* (CPhGs) on the prevention of bovine serum albumin (BSA)-induced hepatic fibrosis in rats. Investigation of the mechanisms of the anti-hepatic fibrosis effect was focused on CPhGs’ influence on the “gut–liver” regulation, including the gut microbiota, intestinal barrier, systemic lipopolysaccharide (LPS) concentration, and LPS-related signaling pathway. The results show that CPhGs restored the diversity of gut microbiota, increased the relative abundance of *Bacteroidetes*, and decreased the relative abundance of *Firmicutes* and *Proteobacteria* in the fibrotic rats. In addition, CPhGs promoted the enrichment of probiotics such as *Blautia*, *Oscillospira*, *Ruminococcus*, *Odoribacter*, *Bacteroides*, and *Parabacteroides* in intestines of these rats. Furthermore, CPhGs reduced histopathological injury in the intestine and restored the tight junctions of the intestine by increasing the expression of ZO-1, occludin, and E-cadherin. CPhGs efficiently reduced serum LPS and liver lipopolysaccharide-binding protein (LBP) levels and inhibited the LPS-TLR4/MyD88/NF-κB pathway, which is related to protein expression in the liver. Correlation analysis confirmed that these beneficial bacteria were negatively associated with pathological damage, while LPS and harmful bacteria were positively associated with liver injury. Our fecal microbiota transplantation (FMT) experiment confirmed that gut microbiota is an important part of disease progression and that CPhGs is useful for the prevention and treatment of hepatic fibrosis. Our data demonstrate that the anti-hepatic fibrosis mechanism of CPhGs was mediated by regulation of the “gut–liver” axis. These results can stimulate consideration for its use in clinical practices.

## 1. Introduction

Liver damage can result from endogenous autoimmune problems and from external factors, such as viral infections, chemical exposure, and misuse of drugs. This damage can progress from hepatitis to liver fibrosis, cirrhosis, and liver cancer, with liver fibrosis playing a central role in disease progression [1]. Liver fibrosis is characterized by compositional changes, excessive deposition of extracellular matrix (ECM), and persistent inflammation that ultimately leads to cirrhosis, liver cancer, and liver failure, which often lead to mortality [2]. Fortunately, liver fibrosis is a reversible pathological process. Therefore, suppressing the continuous inflammatory response and blocking hepatic fibrosis are important tools for controlling the progression of liver disease.

The gut microbiota–liver axis refers to the complex interplay between the gut microbiota and the liver, which is pivotal for maintaining hepatic health [3]. Recent studies have demonstrated that the gut microbiota can have positive and negative influences on liver fibrosis. Among the negative influences are the production of metabolites (which can promote fibrotic processes and the modulation of the immune response) and the alteration of gut permeability, which leads to increased systemic inflammation and endotoxemia [4]. An imbalanced gut microbiota can result in increased production of pro-inflammatory cytokines and lipopolysaccharides (LPSs), LPS-activated Toll-like receptor 4, and a cascade of inflammatory responses via the NF-κB/MyD88 signaling pathway [5]. Moreover, damage to the intestinal barrier can further aggravate the injury response. As a result, amelioration of hepatic fibrosis can possibly be achieved through the restoration of bidirectional crosstalk between the gut and liver. A potential approach is the use of certain forms of traditional Chinese medicine (TCM) and natural products, which are known for their multi-target and multi-pathway therapeutic activities that target the gut–liver axis for improved treatment of liver diseases.

*Cistanche tubulosa*, commonly known as “Rou Cong Rong” in Chinese, is a parasitic herb belonging to the Orobanchaceae family and growing in desert areas. It has a long history of use in traditional medicine, particularly in the treatment of weakness, fatigue, and sexual dysfunction [6]. Chemically, *Cistanche tubulosa* is rich in benzyl alcohol glycosides, iridoid terpenes, and lignans, including phenylethanol glycosides, among which phenylethanol glycosides (CPhGs) are the most important active ingredients [7,8]. Modern pharmacology has shown that CPhGs may possess hepatoprotective, neuroprotective, cardioprotective, anti-oxidation, anti-inflammatory, and immunoprotective effects [9,10]. It is reported that CPhGs and their monomers, echinacoside and acteoside, reduce liver injury through antioxidant, anti-inflammatory and immunomodulatory pathways [11]. Our previous study found that CPhGs possessed a notable anti-hepatic fibrosis effect, and its mechanism of action was related to inhibition of the TGF-β1/smad pathways and activation of HSC cells [12]. Additionally, CPhGs have been shown to positively influence the gut microbiota composition, enhance the abundance of beneficial bacteria, and reduce the abundance of pathogenic strains. This modulation was reported to promote a healthier gut environment and enhance integrity of the gut–liver axis [13]. However, the specific roles of CPhGs in modulating gut health and in preventing liver fibrosis have not been adequately addressed. Hence, this study investigated CPhGs’ impact on the “gut–liver axis” via the gut microbiota, intestinal barrier, and systemic LPS concentration to elucidate their roles in liver fibrosis. Our findings should offer a basis for the utilization of CPhGs in clinical practice.

## 2. Results

### 2.1. CPhGs Enhances Liver Function and Alleviates BSA-Induced Hepatic Fibrosis in Rats

**Figure 1:**
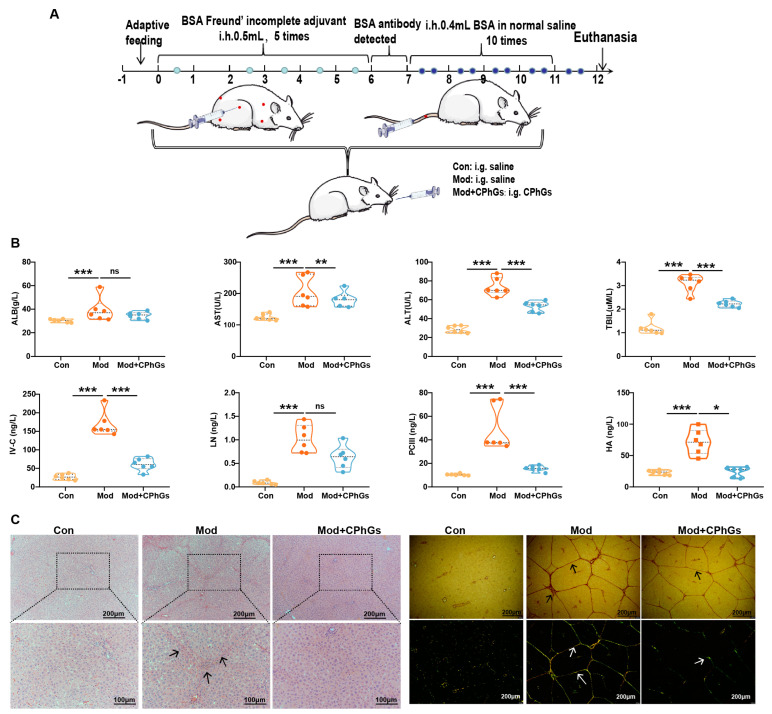
Effect of CPhGs on biochemical indicators and pathological changes in BSA-induced liver fibrosis rats. (**A**) Flowchart of the experimental protocol. (**B**) Changes in serum ALB, ALT, AST, TBIL, HA, PC-III, IV-C, and LN levels. (**C**) Pathological changes (arrows highlight the presence of portal fibrosis; for HE staining, the scale bar represents 100 or 200 µm; and for Sirius red staining, the scale bar represents 200 µm). Data are presented in violin charts, n = 6. * *p <* 0.05, ** *p <* 0.01, and *** *p <* 0.001. ns: not significant.

Our previous study showed that CPhGs exhibited hepatoprotective effects from liver injury and fibrosis [12]. The current study demonstrates that the BSA induction significantly elevated serum ALT, AST, ALB, TBIL, HA, LN, PC III, and IV-C levels (*p <* 0.001); promoted infiltration of inflammatory and abnormal liver cells; induced abnormal liver tissue structure; and increased deposition of collagen fibers, which led to the formation of pseudo-lobules (Figure 1). CPhGs intervention reversed these abnormalities (*p <* 0.01 or *p <* 0.001) and ameliorated pathological changes. Our results clearly show the anti-hepatic fibrosis effect of CPhGs.

### 2.2. CPhGs Improves Abnormal Inflammatory Responses via the TLR4/NF-κB Signaling Pathway

**Figure 2:**
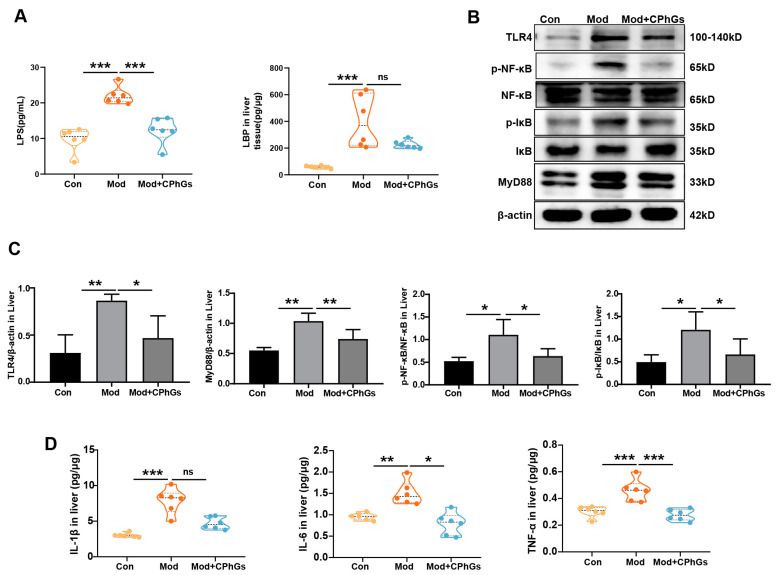
Effects of CPhGs on protein expression of TLR-4/NF-κB in liver tissues. (**A**) changes of the LBP in liver tissue and LPS in serum. (**B**,**C**) expression and quantitative results of proteins related to the TLR-4/NF-κB signaling pathway. (**D**) changes of the TNF-α, IL-6, IL-1β expression in liver tissues. Data were presented as mean ± SD or in violin charts. n = 6, * *p <* 0.05, and ** *p <* 0.01, *** *p <* 0.001. ns: not significant.

Inflammatory response has been known to be an important factor in the progression of chronic liver disease and mediated by extrahepatic substances, e.g., from intestinal and adipose tissue sources [3]. To expand on such observations in our study, expression levels of several proinflammatory cytokines and potential inflammatory substances were examined. Our data indicate that liver from the BSA-treated rats exhibited significant accumulation of IL-1β, IL-6, and TNF-α (Figure 2D, *p <* 0.01 or *p <* 0.001). Additionally, expressions of LPS in serum and LBP in liver were significantly increased compared to the control group (Figure 2A, *p <* 0.001). However, the CPhGs intervention significantly reduced the expression of IL-1β, IL-6, and TNF-α (*p <* 0.05 or *p <* 0.001), and serum LPS and liver LBP levels (Figure 2A, *p <* 0.001). These results indicate that intestinal-derived LPS induced or exacerbated hepatic inflammation and immune response, while CPhGs intervention reduced the levels of LPS and thereby reduced the level of hepatic inflammation. Further analysis revealed that serum LPS levels were positively correlated with the general indicators, except for AST and PC-III indicators (Figure S1). These results validate the point that substances of intestinal origin caused liver inflammation in rats with liver fibrosis.

Previous reports showed that LPS induced host’s response through the TLR4/NF-κB signal pathway, stimulated immune cells to produce many inflammatory cytokines such as IL-1β, IL-6, and TNF-α, and caused excessive activation of the immune system [14]. Our study shows that the TLR4, MyD88, p-IκB, and p-NF-κB protein expressions levels were considerably elevated in rats with BSA-induced liver fibrosis compared to the controls, while the CPhGs intervention effectively inhibited these changes (Figure 2B,C, *p <* 0.05). The results indicate that CPhGs played an anti-hepatic fibrosis role via inhibiting expression of the LPS-TLR4/MyD88/NF-κB signaling pathway.

### 2.3. CPhGs Attenuates Gut Barrier Injury

**Figure 3:**
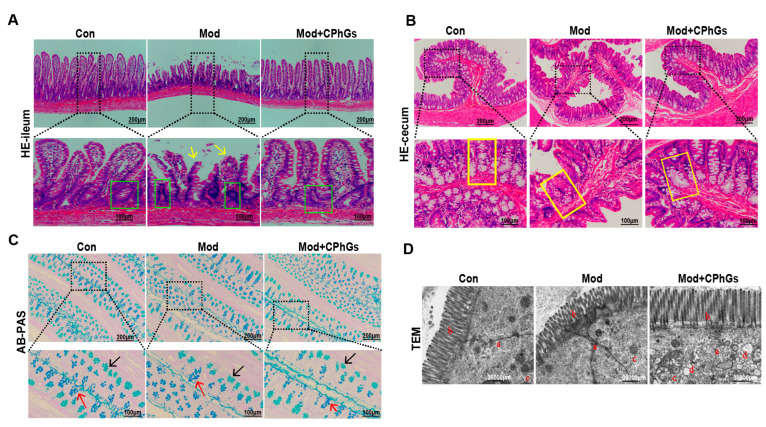
Effects of CPhGs on pathological observations of the intestinal tract. (**A**) effects of CPhGs on histomorphology in the ileum tissues (The yellow arrows point to fragmented intestinal villi; the green boxes circled are intestinal crypts, the scale bar represents 100 or 200 µm). (**B**) effects of CPhGs on histomorphology in the ileum and cecum tissues (The yellow boxes are secretory glands of the cecum, which were almost absent in the model group; the scale bar represents 100 or 200 µm). (**C**) the representative images of AB-PAS staining of intestinal tissues (The black arrows are immature goblet cells; the red arrows are goblet cells that are secreting; the scale bar represents 100 or 200 µm). (**D**) ultrastructural analysis in intestinal tissues by transmission electron microscopy (a: cell gap, b: intestinal villi, c: mitochondria, d: phagosome, at 30,000 µm magnification).

It is well-known that significant increases of LPS in blood were closely related to damage to the intestinal barrier [15]. Thus, key indicators of the gut barrier integrity were further examined in our study. As shown in Figure 3A, the colonic glands in the model group were significantly atrophied and goblet cells were almost nonexistent. After the CPhGs intervention, the ileum and cecum were structurally intact and morphologically normal (Figure 3A). Furthermore, the AB-PAS staining reveals that BSA exposure led to destruction of crypt architecture, decreased crypt density, and depletion of goblet cells, compared to the control group (Figure 3B). Under the electron microscope (Figure 3C), the well-formed tight junctions and preserved organelles in the control group were disrupted in the model group (e.g., swollen mitochondria), while the junctions were restored with CPhGs intervention.

**Figure 4:**
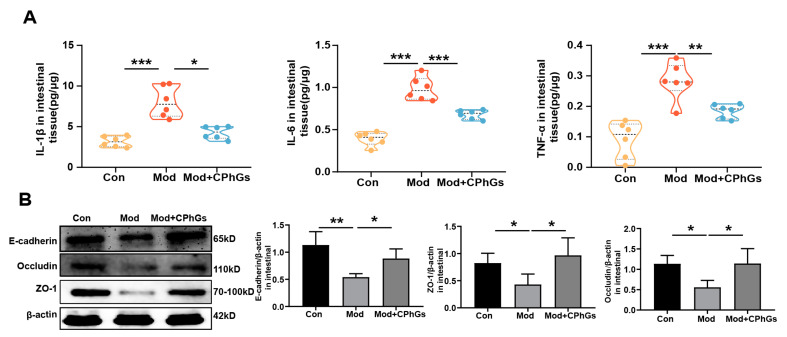
Effects of CPhGs intervention on intestinal barrier defects in rats with BSA-induced liver fibrosis. (**A**) Effects of CPhGs on IL-1β, IL-6, and TNF-α activities of gut tissues in BSA-induced liver fibrosis. (**B**) Effects of CPhGs on intestinal mucosa occludin, ZO-1, and E-cadherin protein expression in rats with BSA-induced liver fibrosis. Data are expressed as mean ± SD or in violin charts, n = 6, * *p <* 0.05, ** *p <* 0.01, and *** *p <* 0.001.

The integrity of tight junction proteins such as ZO-1, occludin, and E-cadherin played a crucial role in maintaining the intestinal barrier by reducing expression of tight junction proteins which greatly increased intestinal permeability [16]. Through a continuous irritation of BSA, our study shows that the intestinal barrier was damaged, as indicated in Figure 4, and there were decreased ZO-1, Occludin, and E-cadherin protein expression levels (*p <* 0.05 or *p <* 0.01). Increased intestinal permeability was reported to be associated with overexpression of inflammatory cytokines (IL-6, IL-1β, TNF-α) in the intestinal epithelium, thereby causing further aggravation of damage [17]. In our study, BSA induced significant increases in TNF-α, IL-6, and IL-1β production in intestinal tissues (*p <* 0.001). After the CPhGs intervention, these indicators were significantly reduced (*p <* 0.05 or *p <* 0.01). Other reports showed that damage to the intestinal barrier and increased intestinal permeability led to augmentation of the serum LPS concentration [14]. Together with our observations, CPhGs showed a crucial role of improving gut permeability by suppressing intestinal inflammation.

### 2.4. Gut Microbiota Analysis

**Figure 5:**
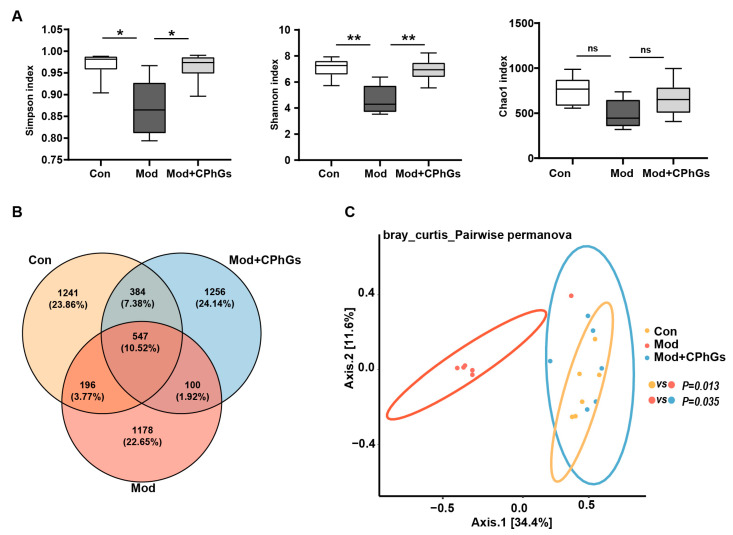
Effects of CPhGs on gut microbiota dysbiosis in rats with BSA-induced liver fibrosis. (**A**) Chao1, Simpson, and Shannon indices analysis was performed to show α-diversity. (**B**) OTU Venn diagram. (**C**) Principal coordinate analysis (PCoA) was performed to show β-diversity. n = 6. * *p <* 0.05, ** *p <* 0.01. ns: not significant.

Perturbation of the gut microbiota has been reported to compromise the integrity of the intestinal barrier [18]. In our study, 16s rDNA sequencing was conducted on gut microbiota in rats to monitor changes in gut flora under different experimental conditions. The sequencing results were organized into operational taxonomic units (OTUs) as indicators for microbiota biodiversity. Using Venn diagrams, 4222 OTUs were detected, and 547 OTUs were common across the three groups; 1247 were specific for the control group, 1178 for the Mod group, and 1256 for the Mod + CPhGs group (Figure 5A,B). It has been recognized that among OTUs, α-diversity indices represent the overall degree of richness and evenness and reflect the microbiome diversity of single samples, including the Chao1 estimator, Simpson and Shannon indices. The Chao1 index represents the richness of microbial species, while the Shannon index and the inverse Simpson index reflect the evenness and richness of microbial communities, focusing, respectively, on rare and dominant microorganisms [19]. In our study, the α-diversity analysis reveals significantly reduced Simpson (*p <* 0.05) and Shannon indices (*p <* 0.01) in the model group compared to the control group, whereas the two indices were significantly improved after CPhGs intervention in the model group (*p <* 0.01).

Alpha-diversity was reported to be an effective indicator for the within-habitat diversity of gut microbiota, whereas the β-diversity indicated variations in microbial communities between samples [20]. Our β-diversity analysis demonstrates differences in the structure of the gut microbiota among the three groups. As shown in Figure 5B, the principal coordinate analysis (PCoA) reveals that the gut microbial composition of BSA-induced liver fibrosis was significantly different from that of the control group (*p <* 0.05). The gut microbial composition of the CPhGs intervention group was significantly different from that of the model group (*p <* 0.05) and had become insignificantly different from the control group. These results indicate that CPhGs interventions improved gut microbial features and enhanced total species richness and diversity.

**Figure 6:**
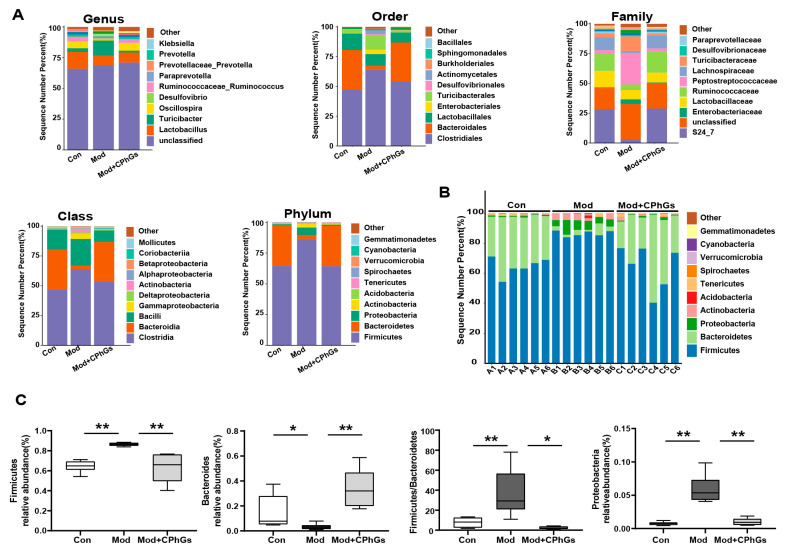
Effect of CPhGs on phylum levels of gut microbiota in rats with BSA-induced liver fibrosis. (**A**) Alterations of the intestinal flora comparison at the phylum, class, order, family, and genus levels. (**B**) Microbiota composition at the phylum level is shown. (**C**) Relative abundance of the dominant phylum of the intestinal flora compared in various groups (*Firmicutes, Bacteroidetes, Proteob*ac*teria*, and *Firmicutes*/*Bacteroidetes*). n = 6, * *p <* 0.05, ** *p <* 0.01.

As shown in Figure 6, the CPhGs intervention induced changes in microbial distribution which were mainly characterized by differential alterations of the gut microbiota at the phylum, class, order, family, and genus levels. As for composition of gut microbiota, the composition was generally determined by two dominant bacterial phyla representing more than 90% of the overall microbial communities (*Firmicutes*, *Bacteroidetes*), followed by smaller contributions from *Proteobacteria* and *Actinobacteria* [21]. In comparison with the control group, our model group showed a significant increase in the relative abundance of *Firmicutes* (*p <* 0.01), and *Proteobacteria* (*p <* 0.01), a decrease in the relative abundance of *Bacteroidetes* (*p <* 0.05), and a significant decrease in the ratio of *Firmicutes* to *Bacteroidetes* (*p <* 0.01), which was effectively normalized by the CPhGs intervention (*p <* 0.05). Correlation analysis showed that *Firmicutes* and *Proteobacteria* were positively correlated with most of the phenotypic indicators. However, *Bacteroidetes* was negatively correlated with serum ALT, TBIL, IL-6, TNF-α, LN, HA, and IL-6 in the liver (*p <* 0.05, *p <* 0.01 or *p <* 0.001, Figure S1).

**Figure 7:**
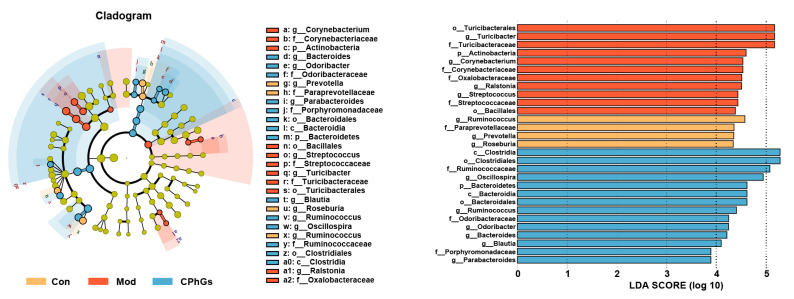
Major species in the intestinal flora of the three groups of rats, identified using the LEfSe analysis. n = 6, LDA > 4.

To investigate the influence of CPhGs on the gut microbiota of hepatic fibrosis in rats, an LEfSe analysis was performed. As illustrated in Figure 7, the abundance of harmful bacteria such as *Corynebacterium*, *Turicibacter*, *Ralstonia*, and *Streptococcus* was enhanced in the model group. After the CPhGs intervention, the abundance of beneficial bacteria such as *Blautia*, *Oscillospira*, *Ruminococcus*, *Odoribacter*, *Bacteroides*, and *Parabacteroides* was increased (Figure 7). These results show that CPhGs effectively reprogrammed the dysbiosis of the gut microbiota in the BSA-induced hepatic fibrosis. Additionally, gut microbiota enriched in the model group, such as *Ralstonia* and *Streptococcus*, were positively related to liver function, liver fibrosis and liver inflammatory indicators. Additionally, gut microbiota enriched in the CPhGs group, such as *Oscillospira*, *Odoribacter*, *Bacteroides*, and *Parabacteroides*, almost negatively correlated to these general indicators. The correlation analyses between the overall indicators and gut microbiota show that CPhGs suppressed liver inflammation and fibrosis by decreasing the prevalence of detrimental bacteria and promoting the growth of beneficial bacteria (*p <* 0.05, *p <* 0.01 or *p <* 0.001, Figure S1).

### 2.5. Preventive Effect of CPhGs on Liver Fibrosis

**Figure 8:**
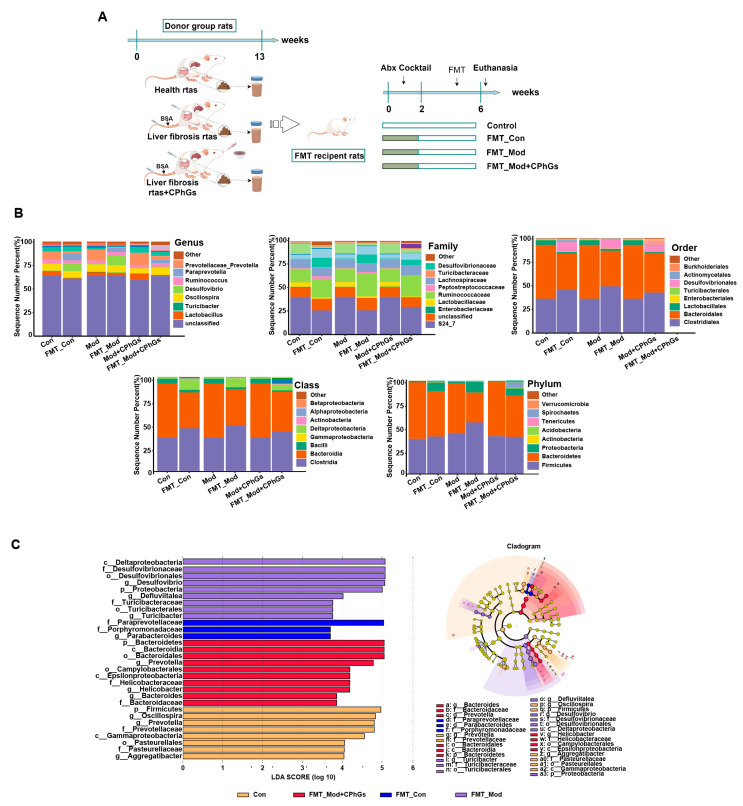
Modeling of gut microbiota in microbiota-depleted rats using the FMT assay. (**A**) Flow chart of FMT experiment. (**B**) Restoration of the intestinal flora at the phylum, class, order, family, and genus levels (n = 6). (**C**) Major species in the intestinal flora identified using LEfSe analysis (n = 6, LDA > 3).

Based on the 16s rDNA analysis, our data indicate the re-establishment of gut microbiota composition in all FMT rats at various taxonomic levels, including phylum, class, order, family, and genus (Figure 8A). Specifically, the evolutionary trends in microbiota of *Firmicutes* (*c/o_Clostridia*, *c/o_Bacteroidia*, *o_Lactobacillale*, *f_Peptostreptococcaceaeet* et al.), *Proteobacteria* (*c/o/f_Deltaproteobacteria* et al.), and *Bacteroidetes* remained consistent both before and after the intervention. This discovery demonstrates that the intervention effectively restored the imbalanced gut flora and modulated the intestinal microecology in bacteria-depleted rats. Further analysis using LEfSe (Figure 8B) shows that three representative OTUs, *f_Paraprevotellaceae*, *g_parabacteria*, and *f_Porphyromnadaceae,* were enriched in the FMT-Con group. Among them, *f_Paraprevotellaceae* was also the core gut microbiota in the control group. Similarly, 9 representative OTUs and 10 representative OTUs were enriched in the FMT_Mod group and FMT_Mod + CPhGs group, respectively, with *g_Turicibacter*, *f_Turicibacteraceae*, and *o_Turicibacterales* identified as critical gut microbiota in both the Mod and FMT_Mod groups and *g_Bacteroides* identified as critical gut microbiota in both the Mod + CPhGs and FMT_Mod + CPhGs groups.

### 2.6. Gut Microbiota of Rats with Liver Fibrosis Transmit the Inflammatory Response and Induce Liver Injury

**Figure 9:**
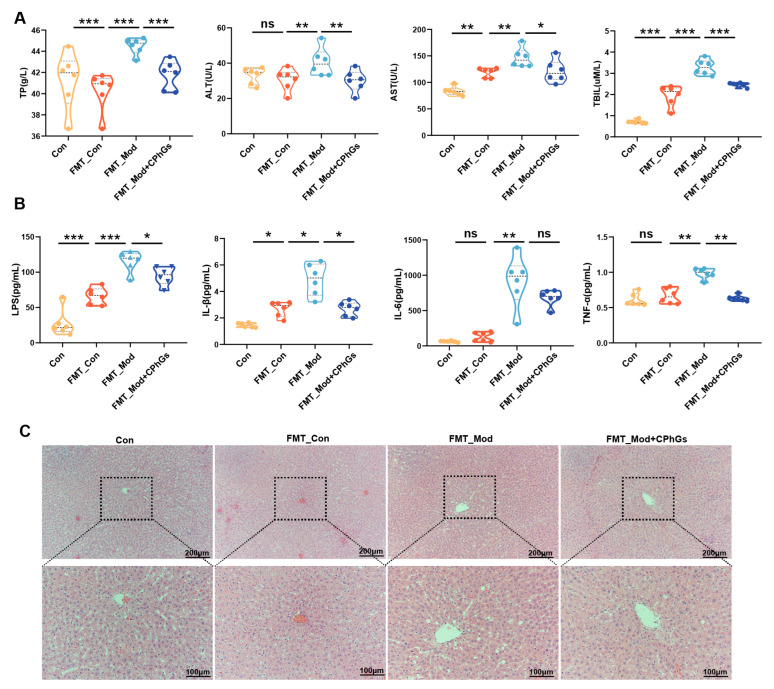
Effects of biochemical indices and pathological changes in FMT receptor rats. (**A**) Changes in serum ALT, AST, TBIL, and TP levels. (**B**) Changes in serum LPS, IL-1β, IL-6, and TNF-α. (**C**) Pathological changes (HE staining, 100×). Data are presented in violin charts, n = 6. * *p <* 0.05, ** *p <* 0.01, *** *p <* 0.001. ns: not significant.

The results depicted in Figure 9 indicate that there were no significant differences between the control group and the FMT_Con group with respect to serum ALT, TBIL, TP, IL-1β, IL-6, and TNF-α levels. Conversely, the administration of fecal suspension from liver-fibrotic rats via gavage to recipient rats resulted in a significant increase in systemic inflammation and liver injury (*p <* 0.05, *p <* 0.01 or *p <* 0.001). Statistical analyses revealed a significant reduction in the levels of ALT, AST, TBIL, IL-1β, IL-6, and TNF-α in the FMT_Mod + CPhGs group compared to the FMT_Mod group (*p <* 0.05). In addition, histological examinations (Figure 9C) show that BSA treatment led to inflammatory cell infiltration and accumulation of abnormal liver tissues in the FMT_Mod rats, while FMT_Mod + CPhGs rats did not display significant liver pathology compared to the FMT_Mod group. These results indicate that CPhGs intervention may have provided a hepatoprotective effect in rats with BSA-induced liver injury, potentially through enhancing the resilience of microbiota. Furthermore, serum LPS was increased in all FMT groups, and serum LPS levels were significantly higher in the FMT_Mod rats than in the FMT_Mod + CPhGs and FMT_Con rats (*p <* 0.05), which again demonstrates that the health status of the donor rats was transferred to the recipient rats via FMT.

## 3. Discussion

Most chronic liver diseases are characterized by liver fibrosis, and it is possible to reduce the outcome of the disease by restricting the progression of liver fibrosis [15]. Studies have shown that several herbs and their extracts have been capable of preventing liver fibrosis [22]. Specifically, CPhGs, the main extract of *Cistanche tubulosa*, was shown to protect liver function, by reducing the secretion of proinflammatory cytokines and reversing hepatic fibrosis [23], as expanded upon in our study.

It has been reported that the progression of chronic liver diseases involves disruption of intestinal homeostasis, and a large proportion of the substances that induced liver inflammation had their source in the intestine [24]. Our investigation extended the previous findings by discovering that the LPS levels were significantly increased in both the serum and liver, and increased serum LPS levels were positively correlated with the progression of liver injury, inflammation, and fibrosis. Studies have shown that increased serum LPS levels are associated with inflammation in extraintestinal organs (such as the liver and kidney) [14]. LPS, which belongs to the TLR4 signaling pathway agonist, activates the TLR4/NF-κB signaling pathway and promotes the secretion of proinflammatory cytokines [25]. These observations substantiate our findings, especially on CPhGs’s inhibition of the activation of the TLR4/NF-κB signaling pathway by BSA. The elevation of LPS levels in the serum and liver indirectly indicates that BSA compromises intestinal epithelial integrity and disrupts intestinal permeability. Conversely, CPhGs pretreatment was found to enhance ileum morphology, reduce intestinal permeability and inflammatory cytokine secretion, and elevate the expression of tight junction proteins, with support from other studies [26]. Thus, CPhGs pretreatment can prevent damage from exposure to certain toxic substances by regulating the functions of the intestinal barrier.

Patients with chronic liver diseases have shown compromised integrity of the intestinal barrier as well as disrupted structure of the gut microbiota [27]. Our observations indicate that hepatic fibrosis in rats is associated with alterations in the composition of the gut microbiota, characterized by an increase in Firmicutes and Proteobacteria, a decrease in Bacteroidetes, and an elevated ratio of *Firmicutes* to *Bacteroidetes*, resulting in heightened proinflammatory markers in the intestine. Intervention with CPhGs was found to reverse these changes. In support of our observations, an increase in Firmicutes contributed to the development of hepatic steatosis and elevation of TNF-α mRNA levels [28], and an elevated ratio of *Firmicutes/Bacteroidetes* was linked to inflammation and oxidative stress [29]. Excessive growth of Firmicutes and Proteobacteria produced metabolic endotoxins, such as LPS, that triggered systemic inflammation and metabolic imbalance [30]. Indeed, a significant increase in the *Firmicutes/Bacteroidetes* ratio in rats with hepatic fibrosis, accompanied by a notable systemic inflammatory response, was observed in our study. Correlation analyses also confirm this conclusion.

Further LEfSe analysis revealed that the guts of rats with induced liver fibrosis were predominantly enriched with conditionally pathogenic bacteria, whereas CPhGs intervention increased the abundance of beneficial bacteria. Correlation analysis confirmed that these harmful or beneficial bacteria were closely associated with the progression or regression of liver disease, respectively. For instance, *Ralstonia* has been reported to be a causative organism with high mortality in fragile populations [31]. In contrast, O*doribacter* was reported to be a key component which promoted both metabolic and immune cell protection [32]. In addition, Parabacteroides alleviated hepatic fibrosis by modulating hepatocyte pyroptosis and bile acid metabolism [33]. On the other hand, *Oscillospira* produced a sufficient protective IgA response against a potential pathobiont and mediated the hepatoprotective effect via regulating the level of oxidative stress [34]. Our data indicate that CPhGs treatment positively influences the composition of intestinal microbiota by enhancing diversity and abundance, promoting the growth of beneficial probiotic bacteria, and reducing harmful bacterial populations. These positive effects are strongly associated with the reversal of liver fibrosis by CPhGs.

Causal relationships between CPhGs in restoring intestinal microecological balance and their potential in reversing liver fibrosis remain, however, unclear. Consequently, a FMT experiment was conducted by us to investigate this relationship. FMT was a useful strategy for treating disease by altering the patient’s gut microbiota through the transplantation of gut microbiota [35]. In our study, the recipient rats displayed a more severe inflammatory response and abnormal liver function, compared to rats in the FMT-Mod + CPhGs group. As shown in our study and as reported, different rat donors with different gut flora compositions induced different phenotypes in recipient rats [36]. To validate this perspective, the colonization of the gut microbiota was examined in the recipient rats. The results revealed that many Proteobacteria colonized the intestines of rats in the FMT_Mod group, causing liver injury and inflammation. However, due to the intervention of CPhGs, which were shown to reduce harmful bacteria and increase beneficial bacteria, the FMT_Mod + CPhGs group of rats did not exhibit severe inflammatory responses and liver damage. The same was true for the FMT_Con group.

Previous reports indicate that liver diseases are characterized by disorders of the “gut–liver axis” and impairment of the intestinal barrier is a prerequisite for alterations in the “gut–liver axis”, with LPS serving as a key gut-derived mediator in “gut–liver” communication for liver disease [37]. In clinical and preclinical studies, exogenous substances such as medicinal plants, grains, and teas have interacted with the gut microbiota and reduced disease risks [34]. Our observations demonstrate that CPhGs intervention attenuated BSA-induced hepatic injury, hepatic inflammation, and hepatic fibrosis. In addition, the intervention improved gut microbiota structure, restored intestinal barrier function, and alleviated intestinal-derived LPS. Accordingly, the signaling pathway of LPS-TLR4/Myd88/NF-κB in liver tissue was suppressed. The data indicate that CPhGs intervention improved liver function and reversed damage in BSA-induced liver fibrosis by regulating the “gut–liver axis” in rats.

## 4. Materials and Methods

### 4.1. Chemicals and Reagents

CPhGs were purchased from the Hetian Di Chen Medical Biotechnology Co., Ltd. in Xinjiang, China. The material was provided in powder form, and the purity of CPhGs was confirmed by high-performance liquid chromatography (HPLC, Figure S1). BSA was purchased from Sigma (St. Louis, MO, USA). Type IV collagen (IV-C), hyaluronidase (HA), type III precollagen (PC-III), LBP, tumor necrosis factor-α (TNF-α), interleukin-6 (IL-6), and interleukin-1β (IL-1β) kits were sourced from Elabscience Biotechnology Co., Ltd. (Wuhan, Hubei, China). The LPS kit was purchased from Jianglai Biotechnology Co., Ltd. in Shanghai, China. The laminin (LN) kit was purchased from ELK Biotechnology Co., Ltd. (Wuhan, Hubei, China). The alanine transaminase (ALT), aspartate transaminase (AST), albumin (ALB), and total bilirubin (TBIL) kits were obtained from Myriad Bio-Medical Electronics Co., Ltd. (Shenzhen, Guangdong, China). The MyD88 antibody, IκB antibody, p-IκB antibody, NF-κB (p65) antibody, p-NF-κB (p-p65) antibody, occludin antibody, and ZO-1 antibody were purchased from Abcam (Cambridge, UK). Ampicillin, metronidazole, vancomycin, and neomycin were acquired from Maikelin Biotechnology Co., Ltd. (Shanghai, China).

### 4.2. Experimental Animals

Healthy adult male Sprague Dawley (SD) rats (180–220 g) were obtained from the Animal Center of Xinjiang Medical University (SCXK2018-0002). The rats received humane care, and the experimental protocol was conducted under the guidelines of the Animal Ethics Committee of the Animal Center of Xinjiang Medical University (IACUC-20220720-28). Rats were housed in an SPF-level experimental environment, which included stable room temperature (23 ± 2 °C) and humidity (55 ± 10%) with a 12 h light/dark cycle and free access to food and water (SYXK2018-0003).

### 4.3. BSA-Induced Hepatic Fibrosis Rat Model and CPhGs Treatments

After one week of housing the SD rats, they were randomly divided into three groups of six rats per group: control (Con), model (Mod), and CPhGs (Mod + CPhGs). Except for the rats in the control group, the remaining rats were used to establish the hepatic fibrosis model. Rats in the Mod + CPhGs group were treated with CPhGs solution (100 mg/mL) per day at a volume of 0.5 mL/100 g. Rats in the model and control groups were administered equal amounts of saline. The intervention with CPhGs began immediately following the completion of the adaptive feeding period and continued for 12 weeks. At the end of the intervention, all rats were sacrificed for the collection of serum and fecal samples and liver and intestinal tissues (Figure 1A).

The hepatic fibrosis rat model was established through a series of steps. First, rats were subcutaneously injected with BSA Freund’s incomplete adjuvant (9 mg/mL) five times with 0.5 mL each, with 14 days between the first two injections and seven days between the remaining three injections. One week later, blood samples were collected from the rat retinal vein plexus and tested for serum albumin antibodies on agar plates. Second, rats that tested positive for BSA antibodies were then injected with 0.4 mL of BSA solution through the tail vein, with the following concentrations: 5.00, 5.25, 5.50, 5.75, 6.00, 6.25, 6.50, 6.75, 7.00, and 8.00 mg/mL. Rats were treated twice a week until all the concentrations of BSA solution had been injected, as previously described [38].

### 4.4. The Fecal Microbiota Transplantation (FMT) Design for Investigation

Preparation of fecal suspension: approximately 100 g of fecal samples were obtained from all the rats in the first phase of the experiment (Con, Mod, and Mod + CPhGs). The samples were resuspended in 500 mL sterile anaerobic PBS and then centrifuged at 700× *g* for 5 min to collect the microbial supernatants. The supernatants were aliquoted into 2 mL portions and stored in a −20 °C freezer.

Rats were randomly assigned to the following four groups: control (Con), FMT control (FMT_Con), FMT model (FMT_Mod), and FMT Model + CPhGs (FMT_CPhGs) groups, with six rats in each group. Before FMT and except for the rats in control group, the remaining rats were treated with a combination of antibiotics (0.5 mL/100 g) for a period of 14 consecutive days, including 1 g/L metronidazole, 1 g/L ampicillin, 1 g/L neomycin, and 0.5 g/L vancomycin, to completely clear their gut microbiota. After the antibiotic intervention, the supernatants were then transplanted into the gut microbiota-depleted rats at a dosage of 2 mL per rat by gavage for a period of 4 weeks [30]. After the intervention (in the seventh week), all rats were sacrificed for the collection of serum and liver and gut tissues (Figure 9A).

### 4.5. Western Blotting

The liver and intestinal tissues were homogenized in RIPA buffer containing Halt Protease Inhibitor Cocktail for 10 min and centrifuged at 10,000× *g* for 10 min. Each sample’s protein concentration was analyzed using a BCA Protein Concentration Determination Kit with the manufacturer’s instructions. Then, the protein sample (20–30 μg) was separated by sodium dodecyl sulfate polyacrylamide gel electrophoresis (SDS-PAGE) and transferred to polyvinylidenefluoride (PVDF) membranes. Next, the PVDF membranes were immersed in fresh nonfat milk (5%) for 2 h at room temperature and curated with primary antibody for a night in the refrigerator (4 °C). These results were visualized by enhanced chemiluminescence (ECL) with chemiluminescence after the membrane had been incubated in the secondary antibodies for 2 h at room temperature the following day. In this experiment, the following ratios of specific primary antibodies were used: TLR4 (1:2000), NF-κB (1:1000), IκB (1:2000), p-NF-κB (1:1000), p-IκB (1:1000), MyD88 (1:2000), zonula occludens-1 (ZO-1, 1:500), Occludin (1:1000), E-cadherin (1:2000), β-actin (1:1000), and secondary antibodies (1:25,000).

### 4.6. Biochemical Assays

Liver function indices including ALT, AST, TBIL, and ALB were detected using a blood biochemical analyzer to assess liver health status according to the manufacturer’s instructions. Additionally, serum or tissue inflammatory factors such as TNF-α, IL-1β, and IL-6 as well as serum hepatic fibrosis factors HA, IVC, LN, and PCIII were detected via an enzyme-linked immunosorbent assay (ELISA) according to standard protocols.

### 4.7. Histopathological Analysis

The liver and intestinal tissue samples were fixed in 10% neutral buffered formalin and embedded in paraffin. Then, 5 μm thick sections in liver tissue were cut with a microtome and stained with hematoxylin–eosin dye and Sirius scarlet dye, and 5 μm thick sections of intestinal tissue were cut with a microtome and stained with hematoxylin–eosin dye and periodic acid Schiff and Alcian blue (AB-PAS) dye. We examined the sections using light microscopy.

### 4.8. Ultrastructural Analysis

Ileum tissue samples were promptly fixed in 3% glutaraldehyde and kept at 4 °C for 4 h. Subsequently, the samples underwent dehydration using a series of ethanol and propylene oxide, followed by embedding in epoxy resin. Thin sections were prepared, stained with uranyl acetate and lead citrate, and observed using transmission electron microscopy to assess the ultrastructure of the tight junctions in the ileum.

### 4.9. 16S rDNA

Rat stool DNA was extracted from the frozen samples using cetyltrimethylammonium bromide (CTAB) methods according to the manufacturer’s instructions. The v3-v4 region of the extracted DNA samples was amplified via PCR techniques with the primer sequences 341F (5′-CCTAYGGGRBGCASCAG-3′) and 806R (5′-GGACTACNNGGGTATCTAAT-3′). Next, the sequence library was constructed via the NEB Next^®^ Ultra DNA Library Prep Kit according to the manufacturer’s instructions, and sequence analysis was performed by a NovaSeq 6000. Then, the successfully combined sequences were subjected to quality control using QIIME2 dada2 https://docs.qiime2.org(accessed on Jan 2019).. Methods such as analysis of composition of microbiomes (ANCOM), one-way analysis of variance (ANOVA), Kruskal–Wallis, LEfSe, and DESeq2 were applied to identify bacteria with differential abundances between groups and samples.

### 4.10. Statistical Analysis

Statistical analysis was performed using SPSS 26.0 and GraphPad Prism 8.0. Normally distributed data are presented as mean ± standard error of the mean (SEM); comparisons between two groups were conducted using Student’s *t*-test, and comparisons among multiple groups were performed using an ANOVA. Non-normally distributed data are expressed as median and interquartile range, and differences between groups were analyzed using non-parametric tests. A *p*-value of less than 0.05 was considered statistically significant.

## 5. Conclusions

Our study demonstrates the significant contribution of the “gut microbiota–liver axis” to the development and recovery of hepatic fibrosis in rats, and the observed mechanism of intervention by CPhGs was via modulating crucial factors of the “gut microbiota–liver axis”. There are some limitations in our study which should be described. It is well known that various factors of the”gut microbiota–liver axis” influence each other. Although our study illustrated changes in these key factors which were impacted by CPhGs, specific mechanisms of action need to be further investigated. It is well known that there is a symbiotic relationship among the comoponents of the gut microbiota. Our data show the “prebiotic-like” effect of CPhGs, but the most critical gut bacteria responsible for this effect have not yet been screened out. Finally, additional research is warranted to explore the influence of gut microbiota on the constituents of CPhGs, such as echinacoside and acteoside, and their anti-hepatic fibrosis properties, to comprehensively uncover the pharmacodynamic basis of CPhGs.

## Data Availability

The raw data supporting the conclusions of this article will be made available by the authors without undue reservation.

## Supplementary Materials

The following supporting information can be downloaded at: https://www.mdpi.com/article/10.3390/ph17091149/s1, Figure S1. Correlation analysis of key flora screening and general indicators for each group. * *p* < 0.05, ** *p* < 0.01, *** *p* < 0.001. Figure S2. CPhGs Ingredient Analysis. Figure S3. Effects of CPhGs on proteins expression of TLR4, MyD88, p-NF-κB and p-IκB of liver tissue in BSA-induced hepatic fibrosis rats. Figure S4. Effects of CPhGs on proteins expression of ZO-1, Occludin, and E-cadherin of intestinal tissue in BSA-induced hepatic fibrosis rats. Table S1. Effect of CPhGs on serum ALT, AST, ALB, and TBIL in BSA-induced hepatic fibrosis rats. Table S2. Effect of CPhGs on serum HA, LN, PCIII, and IV-C in BSA-induced hepatic fibrosis rats. Table S3. Effects of CPhGs on the serum LPS and liver LBP expression level. Table S4. Effects of CPhGs on TLR4, MyD88, p-NF-κB, and p-IκB protein expression in BSA-induced hepatic fibrosis rats. Table S5. Effects of CPhGs on the IL-1β, IL-6, and TNF-α expression level in liver tissue. Table S6. Effects of CPhGs on the IL-1β, IL-6, and TNF-α expression level in intestinal tissue. Table S7. Effects of CPhGs on ZO-1, occludin and E-cadherin protein expression in BSA-induced hepatic fibrosis rats. Table S8. Effects of CPhGs on the gut microbiota dysbiosis of alpha diversity index. Table S9. The composition of gut microbiota analysis on phylum level. Table S10. Effects of biochemical indices and pathological changes in FMT receptor rats. Table S11. Effects of biochemical indices and pathological changes in FMT receptor rats

## Abbreviations

ALT, alanine aminotransferase; AST, aspartate aminotransferase; ABX, antibiotics; ANOVA, analysis of variance; ANCOM, analysis of composition of microbiomes; AB-PAS, periodic acid Schiff and Alcian blue; BSA, bovine serum albumin; CPhGs, phenylethanol glycoside from *Cistanche tubulosa*; CTAB, cetyltrimethylammonium bromide; CLD, chronic liver disease; ELISA, enzyme-linked immunosorbent assay; ECL, enhanced chemiluminescence; ECM, extracellular matrix; FMT, fecal transplantation experiments; GAPDH, glyceraldehyde-3-phosphate dehydrogenase; H&E, hematoxylin and eosin; HA, hyaluronidase; HCC, hepatocellular carcinoma; IL-1β, interleukin-1β; IL-6, interleukin-6; IV-C, collagen-IV; DNA, deoxyribo nucleic acid; 16s rDNA, 16S ribosomal DNA; LN, laminin; LBP, lipopolysaccharide binding protein; LPS, lipopolysaccharide; MyD88, myeloid differentiation factor 88; NF-κB, nuclear factor-kappa B; OUT, operational taxonomic unit, PhGs, phenylethanol glycoside; PVDF, polyvinylidene difluoride; PCoA, principal coordinate analysis; PC-III, procollagen type III; SDS-PAGE, sulfate/polyacrylamide gel electrophoresis; SD, Sprague–Dawley; SPF, specific pathogen free; TBIL, total bilirubin; TP, total protein; TNF-α, tumor necrosis factor-α; TLR4, Toll-like receptor 4; TCM, traditional Chinese medicine; TGF-β1, transforming growth factor-beta; WB, Western blot; ZO-1, zona occludens 1.
