# Supplementary material for: Phenylethanol Glycoside from Cistanche tubulosa Attenuates BSA-Induced Liver Fibrosis in Rats by Modulating the Gut Microbiota–Liver Axis
Source: Pharmaceuticals (Basel). 2024 Aug 30;17(9):1149. doi: 10.3390/ph17091149 (PMC11435394; doi:10.3390/ph17091149)
Supplement: Supplementary file 1 [file pharmaceuticals-17-01149-s001.zip › pharmaceuticals-3124290-supplementary.pdf]

Supplementary Materials

This supplementary materials file contains the following contents:

Supplementary Tables	Table S1 to S11
Supplementary Figures	Figures S1 to S3

Table S1. Effect of CPhGs on serum ALT, AST, ALB, and TBIL in BSA-induced hepatic fibrosis rats

Group	ALT(U/L)	AST(U/L)	ALB(g/L)	TBIL(μM/L)
Control	28.58±3.73	124.3±10.24	30.25±1.44	1.97±0.30
Model	73.73±9.47###	204.85±47.85###	39.40±10.13###	3.09±0.38###
Mod+CPhGs	52.95±5.57***	180.93±24.24**	34.62±3.14	2.23±0.14***

Data are presented as mean±SD., *n*=6.###*P*<0.001 versus control group, ***P*<0.01 and ****P*<0.001 versus model group.

Table S2. Effect of CPhGs on serum HA, LN, PCIII, and IV-C in BSA-induced hepatic fibrosis rats

Group	HA (ng/L)	LN (ng/L)	PCIII (ng/L)	IV-C (ng/L)
Control	14.07±2.99	49.12±11.13	10.87±1.78	30.76±7.08
Model	81.81±39.97##	1138.99±497.58##	53.87±17.48###	223.37±117.92###
Mod+CPhGs	36.48±12.44*	448.83±205.39*	22.97±6.88**	102.97±34.70**

Data are presented as mean±SD., *n*=6. ###*P*<0.01, ###*P*<0.001 versus control group, **P*<0.05 and ***P*<0.01 versus model group.

Table S3. Effects of CPhGs on the serum LPS and liver LBP expression level

Group	LPS (pg/mL)	LBP (pg/μg)
Control	493.39±98.23	0.07±0.03
Model	913.72±110.20###	0.57±0.20###
Mod+CPhGs	614.90±26.51*	0.29±0.12**

Data are presented as mean±SD., *n*=6. ###*P*<0.001 versus control group, **P*<0.05 and ***P*<0.01 versus model group.

Table S4. Effects of CPhGs on TLR4, MyD88, p-NF-κB, and p-IκB protein expression in BSA-induced hepatic fibrosis rats

Group	TLR4/β-actin	MyD88/β-actin	p-NF-κB/NF-κB	p-IκB/IκB
Control	0.31±0.19	0.55±0.05	0.53±0.08	0.50±0.16
Model	0.87±0.07##	1.04±0.13##	1.11±0.34#	1.21±0.40#
Mod+CPhGs	0.47±0.24*	0.74±0.16**	0.64±0.16*	0.66±0.34*

Data are presented as mean±SD., *n*=4. #*P*<0.05, ##*P*<0.01 versus control group, **P*<0.05 and ***P*<0.01 versus model group.

Table S5. Effects of CPhGs on the IL-1β, IL-6, and TNF-α expression level in liver tissue

Group	IL-1β (pg/μg)	IL-6 (pg/μg)	TNF-α (pg/μg)
Control	6.50±2.75	0.08±0.02	0.35±0.04
Model	46.27±32.26 ###	0.41±0.07 ###	0.78±0.24 ###
Mod+CPhGs	19.32±4.93*	0.22±0.08***	0.50±0.09**

Data are presented as mean±SD., n=6. ###P<0.001 versus control group, *P<0.05，**P<0.01 and ***P<0.001 versus model group.

Table S6. Effects of CPhGs on the IL-1β, IL-6, and TNF-α expression level in intestinal tissue

Group	IL-1β (pg/μg)	IL-6 (pg/μg)	TNF-α (pg/μg)
Control	3.17±0.66	0.39±0.08	0.09±0.06
Model	8.05±1.90###	0.99±0.14###	0.28±0.06###
Mod+CPhGs	4.26±0.79**	0.38±0.05**	0.18±0.02*

Data are presented as mean±SD., n=6. ###P<0.001 versus control group, *P<0.05 and **P<0.01 versus model group.

Table S7. Effects of CPhGs on ZO-1, occludin and E-cadherin protein expression in BSA-induced hepatic fibrosis rats

Group	ZO-1/β-actin	occludin/β-actin	E-cadherin/β-actin
Control	0.83±0.18	1.13±0.20	1.13±0.24
Model	0.43±0.19#	0.56±0.17#	0.54±0.06##
Mod+CPhGs	0.97±0.32*	1.14±0.37*	0.88±0.18*

Data are presented as mean±SD., n=4. ##P<0.05, ##P<0.01 versus control group, *P<0.05 versus model group.

Table S8. Effects of CPhGs on the gut microbiota dysbiosis of alpha diversity index

Group	chao1	simpson	shannon
Control	767(627,825)	0.981(0.976,0.987)	7.26(6.92,7.48)
Model	445(373,589)	0.865(0.823,0.908) #	4.30(3.81,5.28) ##
Mod+CPhGs	651(551,711)	0.974(0.966,0.984) *	6.94(6.69,7.19) **

Data are expressed in quartiles, n=6. #P<0.05, ##P<0.01 versus control group, *P<0.05 and **P<0.01 versus model group.

Table S9. The composition of gut microbiota analysis on phylum level

Group	Firmicutes	Bacteroidetes	Proteobacteria	Firmicutes/ Bacteroidetes
Control	0.65(0.63,0.68)	0.078(0.059,0.206)	0.0078(0.0059,0.0084)	8.03 (3.67,11.68)
Model	0.86(0.85,0.88) ##	0.030(0.020,0.034) #	0.0535(0.0445,0.0635) ##	29.29(,25.03,44.83) ##
Mod+CPhGs	0.66(0.56,0.74) **	0.320(0.234,0.403) **	0.0093(0.0058,0.0129) **	2.06(1.43,3.29) *

Data are expressed in quartiles, n=6. #P<0.05, ##P<0.01 versus control group, *P<0.05 and **P<0.01 versus model group.

Table S10. Effects of biochemical indices and pathological changes in FMT receptor rats

Group	ALT(U/L)	AST(U/L)	TP(g/L)	TBIL(μM/L)
Con	28.47±4.67	109.70±8.92	41.27±2.68	2.09±0.65
FMT_Con	28.45±4.86	125.52±9.70$^{\triangle}$	41.75±0.07	2.37±0.10
FMT_Mod	49.52±11.88$^{\#\#\#}$	149.68±12.52$^{\#\#}$	44.13±0.94$^{\#\#}$	3.19±0.62$^{\#}$
FMT_Mod+CPhGs	32.43±7.64 **	125.55±15.90 **	43.08±2.28	2.61±0.20 *

Data were presented as mean±SD., n=6. $^{\triangle\triangle}$*P<0.01*, $^{\triangle\triangle\triangle}$*P<0.001* versus control group; $^{\#}$*P<0.05*, $^{\#\#}$*P<0.01*, $^{\#\#\#}$*P<0.001* versus FMT_Con group; **P<0.05*, ***P<0.01*, ****P<0.001* versus FMT_Mod group.

Table S11. Effects of biochemical indices and pathological changes in FMT receptor rats

Group	LPS (pg/mL)	IL-1β (pg/μg)	IL-6 (pg/μg)	TNF-α (pg/μg)
Con	27.46±18.98	0.10±0.02	0.38±0.34	0.10±0.04
FMT_Con	66.16±11.87$^{\triangle\triangle\triangle}$	0.09±0.02$^{\triangle\triangle}$	0.11±0.04	0.41±0.17
FMT_Mod	115.41±14.57$^{\#\#\#}$	0.31±0.16$^{\#\#\#}$	1.81±0.56$^{\#\#}$	3.37±2.35$^{\#\#\#}$
FMT_Mod+CPhGs	94.94±13.20 *	0.15±0.08 ***	1.03±0.53 *	1.18±0.49 ***

Data were presented as mean±SD., n=6. $^{\triangle\triangle}$*P<0.01*, $^{\triangle\triangle\triangle}$*P<0.001* versus control group; $^{\#}$*P<0.05*, $^{\#\#}$*P<0.01*, $^{\#\#\#}$*P<0.001* versus FMT_Con group; **P<0.05*, ***P<0.01*, ****P<0.001* versus FMT_Mod group.

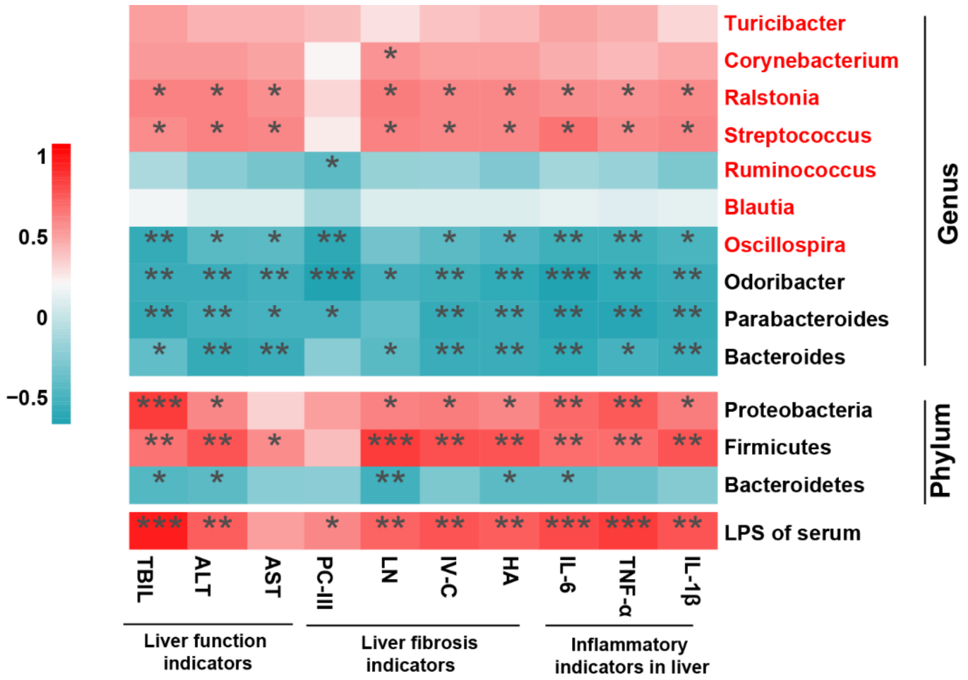

Figure S1. Correlation analysis of key flora screening and general indicators for each group. * *p < 0.05*, ** *p < 0.01*, *** *p < 0.001*.

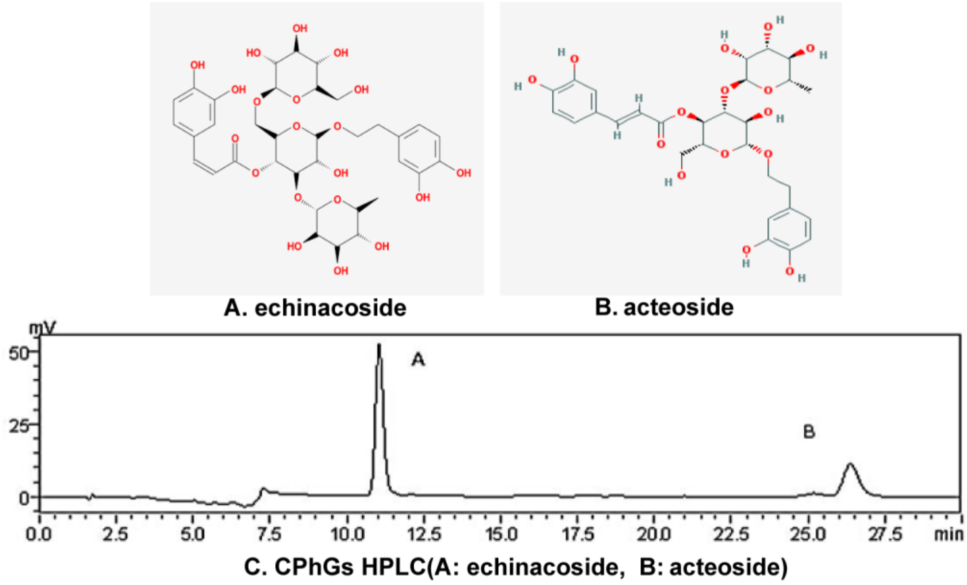

Figure S2. CPhGs Ingredient Analysis.

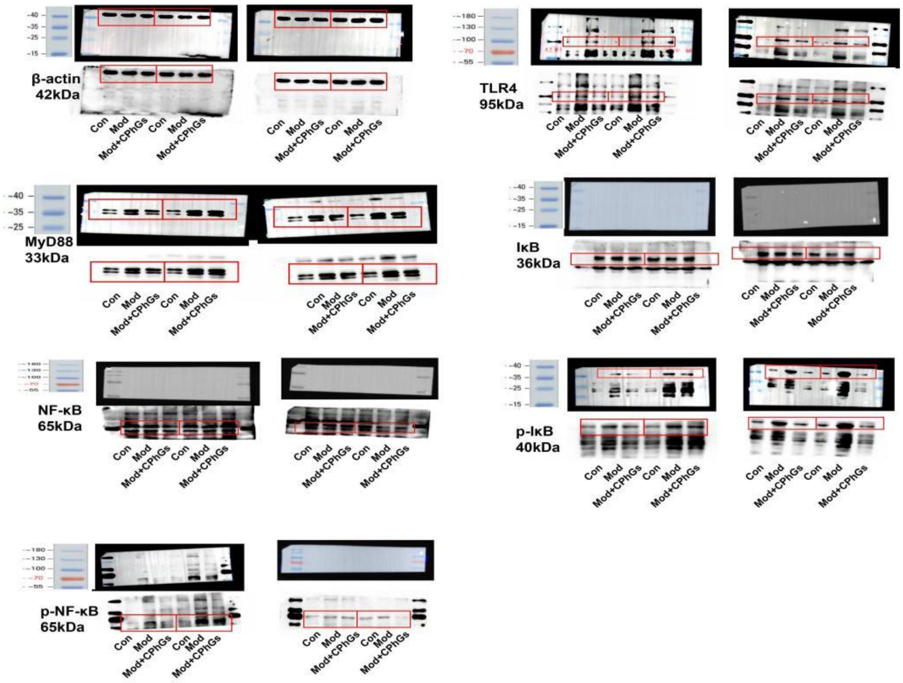

Figure S3. Effects of CPhGs on proteins expression of TLR4, MyD88, p-NF-κB and p-IκB of liver tissue in BSA-induced hepatic fibrosis rats.

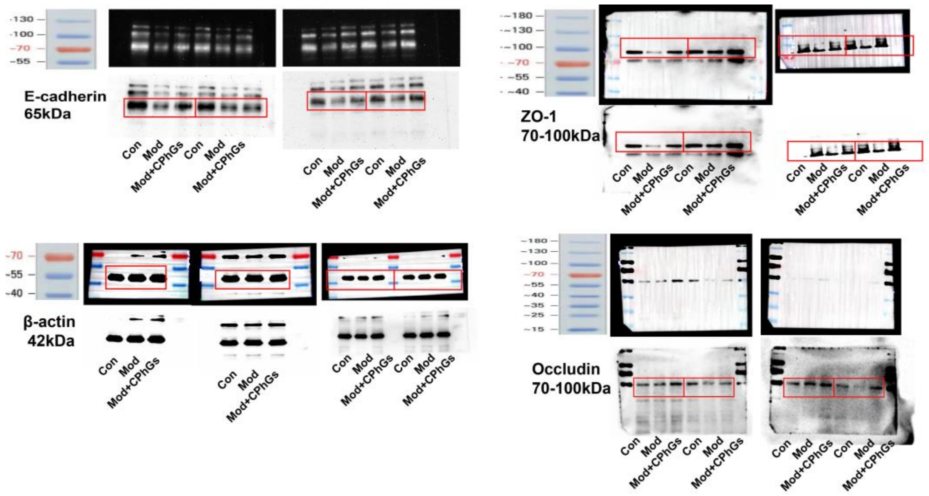

Figure S3. Effects of CPhGs on proteins expression of ZO-1, Occludin, and E-cadherin of intestinal tissue in BSA-induced hepatic fibrosis rats**.**
